# An Effective MM/GBSA Protocol for Absolute Binding Free Energy Calculations: A Case Study on SARS-CoV-2 Spike Protein and the Human ACE2 Receptor †

**DOI:** 10.3390/molecules26082383

**Published:** 2021-04-20

**Authors:** Negin Forouzesh, Nikita Mishra

**Affiliations:** 1 Department of Computer Science, California State University, Los Angeles, CA 90032, USA; 2 Department of Chemistry and Biochemistry, California State University, Los Angeles, CA 90032, USA; nmishra2@calstatela.edu

**Keywords:** binding free energy, implicit solvent, SARS-CoV-2, entropy

## Abstract

The binding free energy calculation of protein–ligand complexes is necessary for research into virus–host interactions and the relevant applications in drug discovery. However, many current computational methods of such calculations are either inefficient or inaccurate in practice. Utilizing implicit solvent models in the molecular mechanics generalized Born surface area (MM/GBSA) framework allows for efficient calculations without significant loss of accuracy. Here, GBNSR6, a new flavor of the generalized Born model, is employed in the MM/GBSA framework for measuring the binding affinity between SARS-CoV-2 spike protein and the human ACE2 receptor. A computational protocol is developed based on the widely studied Ras–Raf complex, which has similar binding free energy to SARS-CoV-2/ACE2. Two options for representing the dielectric boundary of the complexes are evaluated: one based on the standard Bondi radii and the other based on a newly developed set of atomic radii (OPT1), optimized specifically for protein–ligand binding. Predictions based on the two radii sets provide upper and lower bounds on the experimental references: $-14.7\left(\Delta G_{bind}Bondi\right)<-10.6\left(\Delta G_{bind}Exp.\right)<-4.1\left(\Delta G_{bind}OPT1\right)$ kcal/mol. The consensus estimates of the two bounds show quantitative agreement with the experiment values. This work also presents a novel truncation method and computational strategies for efficient entropy calculations with normal mode analysis. Interestingly, it is observed that a significant decrease in the number of snapshots does not affect the accuracy of entropy calculation, while it does lower computation time appreciably. The proposed MM/GBSA protocol can be used to study the binding mechanism of new variants of SARS-CoV-2, as well as other relevant structures.

## 1. Introduction

Emerging as a global threat to human health, the SARS-CoV-2 virus that causes the COVID-19 disease has been widely studied since early 2020 [1]. This fast-growing pandemic highlights the role of computational structural biology and computer-aided drug design (CADD), which have the ability to accelerate the slow and expensive process of drug discovery [2]. Recent computational studies on SARS-CoV-2 have been able to identify frequent points of contact between the virus and ACE2 and the favored conformations of the virus, elucidating potential targets for drug therapy [3,4,5,6]. In structure-based drug discovery, the accuracy and speed of binding free energy estimations of drug-like compounds (ligands) to target biomolecules plays a key role in virtual screening of drug candidates [7,8,9]. Despite decades of research, efficient and accurate computational prediction of binding free energies is still a challenge [10,11,12,13,14]. In theory, the binding free energy of a molecular system can be estimated directly from thermodynamic first principles [15]. However, for any realistic molecular system, approximations must be made to make the estimate computationally feasible. For example, alchemical methods [16,17], simulate changes in the free energy along a pathway that sometimes reflects non-physical properties or, literally, “alchemy”. The required sample points along the pathway are generated via Monte Carlo (MC) or molecular dynamics (MD) simulations. Some of the popular methods in this class are thermodynamic integration (TI) and free-energy perturbations (FEP) [18]. However, these simulations are still computationally expensive, especially when it comes to absolute binding free energy calculation of large protein-ligand complexes [19] and high-throughput virtual screening of thousands of potential drugs [20].

Remarkably more efficient, end-point free energy methods ignore details of the binding pathway and estimate free energy on an ensemble of snapshots representing the bound and unbound states only. These snapshots can be generated by an MD simulation. Molecular mechanics Poisson–Boltzmann surface area (MM/PBSA) and molecular mechanics generalized Born surface area (MM/GBSA) [21,22,23,24] are among the most popular of such methods. While calculations based on practical implicit solvation models, such as Poisson–Boltzmann (PB) or generalized Born (GB), are arguably not as accurate as corresponding estimates based on the best available explicit solvent models, the use of implicit solvent not only brings about computational efficiency but also provides a transparent context for reasoning about the physical origins of observed effects in protein–ligand interactions [25,26,27]. As another important advantage, it is possible to decompose the total free energy into sub-components through MM/PB(GB)SA and measure their contributions separately [8,28]. This feature is certainly useful when it comes to comparing several different free-energy methods. MM/PB(GB)SA is applicable to a wide range of structures [29], from small host–guest systems to large protein–protein complexes with thousands of atoms [8]. This method is mainly used in docking projects where a quick estimate of binding affinities is required [30]. Docking software [31,32] and servers [33,34] rank the feasible poses of a ligand in a binding pocket based on a scoring function in which binding affinity plays an important role. MM/PB(GB)SA can improve the accuracy of these scoring functions on-the-fly. For example, in [35], MM/GBSA was employed to improve the accuracy of docking software in the Drug Design Data Resource (D3R) Grand Challenge 4 (GC4). In another relevant study [36], MM/GBSA demonstrated accurate pose prediction on a large benchmark of protein–ligand complexes with non-redundant binding poses. Recently, MM/GBSA was used to study the effect of nelfinavir stereoisomers on the SARS-CoV-2 main protease [37].

Entropy calculation plays a key role in characterizing the absolute binding free energy within MM/PB(GB)SA methods [38]. Normal-mode analysis (NMA) [39] is a widely used method with promising convergence in calculating configurational entropy [28]. The main drawback of this method is the computational cost, which becomes intractable for large structures due to the expensive calculation of the covariance matrix of internal coordinates for all degrees of freedom. Due to the complex and time-consuming calculations required, the entropy term has been simply ignored in many studies, which leads to considerable overestimation of binding free energy. To tackle this problem, one standard approach is to truncate the protein–ligand complex so that the binding interface remains preserved [40], allowing for a suitable structure size for NMA. The common implementation of this idea is to retain the ligand and remove all protein residues that are more than 8–16 Å  far from the center of mass of the ligand.

**Figure 1:**
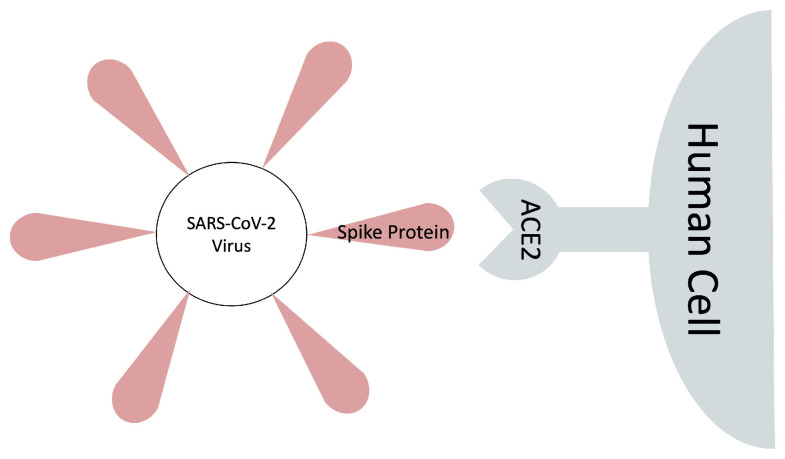
Binding scheme of the SARS-CoV-2 spike protein to the human ACE2 receptor.

In this work, we employ MM/GBSA implemented in AmberTools18 [41] for the binding free energy calculation of the SARS-CoV-2 spike receptor-binding domain (SARS-CoV-2 S RBD) and the human ACE2 receptor complex (PDB ID: 6M0J [42]), see Figure 1. Through the MM/GBSA approach, the absolute binding free energy of a complex is calculated as the sum of gas-phase energy, solvation free energy, and entropic contributions averaged over several snapshots extracted from the main MD trajectory. A grid-based surface GB model is used for estimating the polar component of solvation-free energy, coupled with a new water model and atomic radii introduced earlier in   [43,44]. Human H-Ras and the Ras-binding domain of C-Raf1, the so-called Ras–Raf complex [8,28], is chosen as the reference for the initial evaluation of the MM/GBSA model. As a new extension to our previous works [45,46], a novel truncation strategy is introduced and tested on Ras–Raf and SARS-CoV-2 S RBD/ACE2. Through this strategy, the truncated structure will be one connected component that is biologically more interpretable than the standard truncation methods. The final results are compared with experimental values. The main goal of the work is to assess the potential of the simple and efficient MM/GBSA method to future studies of SARS-CoV-2 binding to the human ACE2 receptor. With respect to emerging mutations of this virus around the world [47,48,49], an efficient computational framework to study binding mechanisms of new variants is vital.

## 2. Materials and Methods

### 2.1. Binding Free Energy Decomposition

**Figure 2:**
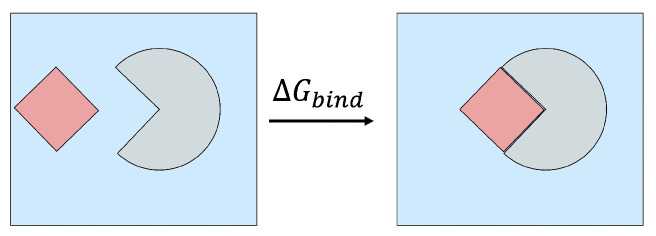
Binding a ligand (shown in red) to a protein receptor (shown in grey) in a box of solvent (shown in blue) releases free energy of $\Delta G_{bind}$. A negative value of $\Delta G_{bind}$ indicates that spontaneous binding occurs, and the magnitude of $\Delta G_{bind}$ characterizes the binding strength (affinity).

Binding-free energy, $\Delta G_{bind}$, of a molecular system is calculated as follows

(1) $$\begin{array}{c} \begin{array}{c} \Delta G_{bind}=\Delta H-T\Delta S, \end{array} \end{array}$$

where ΔH is the enthalpy change in the system, *T* is the absolute temperature in K, and ΔS is the entropy change in the system. A high-level illustration of $\Delta G_{bind}$ between bound and unbound states of a solvated complex is shown in Figure 2.

**Figure 3:**
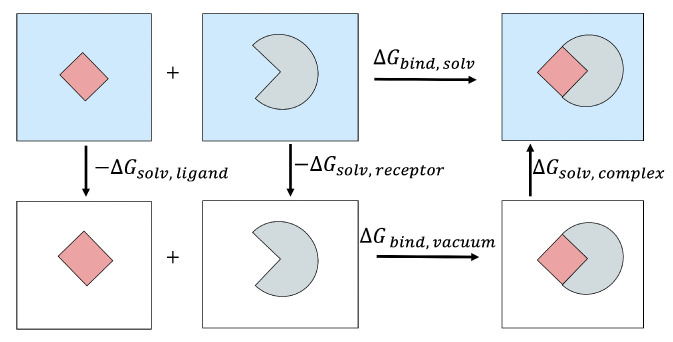
The thermodynamic cycle used to estimate the binding free energy of a protein–ligand complex in the solvent.

In computational studies, a useful way of calculating $\Delta G_{bind}$ is through a thermodynamic cycle shown in Figure 3. With this approach, $\Delta G_{bind,solv}$ is calculated as follows:

(2) $$\begin{array}{c} \begin{array}{c} \Delta G_{bind,\;solv}=\Delta G_{bind,\;vacuum}+\Delta G_{solv,\;complex}-\\ \left(\Delta G_{solv,\;ligand}+\Delta G_{solv,\;receptor}\right) \end{array} \end{array}$$

The solvation-free energy, $\Delta G_{solv}$, is broken into the polar and non-polar components

(3) $$\begin{array}{c} \Delta G_{solv}=\Delta G_{pol}+\Delta G_{nonpol}. \end{array}$$

The free energy in vacuum, $\Delta G_{vacuum}$, is decomposed into the gas-phase energy ($\Delta E_{MM}$) and the configurational entropy of the solute (TΔS)

(4) $$\begin{array}{c} \Delta G_{vacuum}=\Delta E_{MM}-T\Delta S. \end{array}$$

Note that the TΔS above does not exactly correspond to TΔS in Equation (1); specifically, the entropy of solvent re-arrangement [25,27] is subsumed into $\Delta G_{solv}$, which is then considered a part of ΔH. Combining the free-energy components defined above, we obtain $\Delta H=\Delta E_{MM}+\Delta G_{pol}+\Delta G_{nonpol}$. Our approaches for calculating $\Delta G_{solv}$, $\Delta E_{MM}$ and TΔS are explained in Section 2.3, Section 2.4 and Section 2.5, respectively.

### 2.2. MM/PB(GB)SA Free Energy Methodology

**Figure 4:**
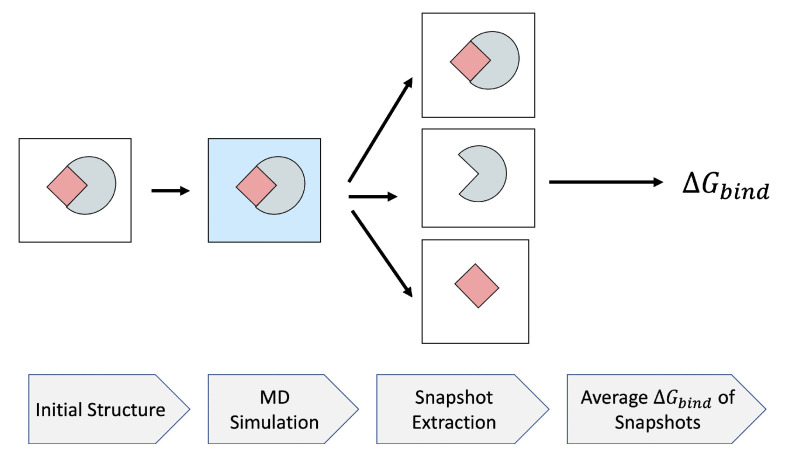
MM/PB(GB)SA flowchart. The initial structure of the complex is solvated using a water model. An MD simulation is run, from which a relatively large number of snapshots are extracted. After removing solvent molecules, the average binding free energy of the snapshots is assigned as the $\Delta G_{bind}$ of the system. The mean and standard deviation of each component of the $\Delta G_{bind}$ are supplemented by MM/PB(GB)SA.

MM/PB(GB)SA is a popular end-point free energy method which estimates $\Delta G_{solv}$ by Poisson–Boltzmann (PB) or generalized Born (GB) implicit solvent model [50], while components of $\Delta E_{MM}$ are estimated based on a classical molecular mechanics force-field. Through the MM/PB(GB)SA approach, the average of $\Delta G_{solv}$ is calculated on a collection of snapshots extracted from an MD simulation. Several decisions have to be made when applying the approach in practice. First, the computational protocol must be selected between the “single-trajectory” (one trajectory of the complex), or “separate-trajectory” (three separate trajectories of the complex, receptor and ligand). In this study, we choose the “single-trajectory” protocol, as it was shown [51] to not only be much faster than the alternative, but also less “noisy” due to the cancellation of intermolecular energy contributions. This protocol applies to cases where significant structural changes upon binding are not expected. The single-trajectory MM/PB(GB)SA begins with the initial structure of the complex in a vacuum. After solvating the structure in a solvent model, an MD simulation is performed to generate the snapshots for further analysis. Then, a relatively large number (typically N>100) of uncorrelated snapshots are extracted to represent the structural ensemble. The binding free energies of these structures are calculated in the implicit solvent after removing the explicit solvent molecules. The average binding free energy over these snapshots is reported as the final $\Delta G_{bind}$. This process is depicted in Figure 4.

With the single-trajectory protocol, the binding free energy of a protein–ligand complex is formally calculated as follows

(5) $$\begin{array}{c} \Delta G_{bind}=<G^{complex}\left(i\right)-G^{protein}\left(i\right)-G^{ligand}\left(i\right){>}_{i}, \end{array}$$

where $<...{>}_{i}$ denotes an average over *i* snapshots extracted from the main MD trajectory. The implementation of this protocol is available in AmberTools18 in Perl [51] and Python [52]. In this work, the former is used to maintain consistency with the reference study [28] opted for tuning the MM/GBSA model.

### 2.3. Solvation Free Energy

#### 2.3.1. Polar Component

A more computationally efficient alternative to the PB, the GB implicit solvent model [53,54], can be used for computing $\Delta G_{solv}$. Generally speaking, GB models have shown to be less computationally expensive than the PB models, although the deterioration of the accuracy has always been a concern. A grid-based surface GB model called GBNSR6 [55] is employed. In a recent study [56], GBNSR6 was shown to be the most accurate among several GB models in terms of approximating $\Delta G_{pol}$ relative to the numerical PB. In this work, $\Delta G_{pol}$ is calculated with the ALPB modification [57,58] (enforcing correct dependence on dielectric constants) of the generalized Born [59] model

(6) $$\begin{array}{c} \begin{array}{cc} \Delta G_{pol} & =\sum_{ij}\Delta G_{ij}^{pol}\approx -\frac{1}{2}\left(\frac{1}{\epsilon_{\mathrm{in}}}-\frac{1}{\epsilon_{\mathrm{out}}}\right)\frac{1}{1+\beta \alpha }\sum_{ij}q_{i}q_{j}\left(\frac{1}{f_{ij}^{\mathrm{GB}}}+\frac{\alpha \beta }{A}\right), \end{array} \end{array}$$

where $\epsilon_{\mathrm{in}}=1$ and $\epsilon_{\mathrm{out}}=80$ are the dielectric constants of the solute and the solvent, respectively, $\beta =\epsilon_{\mathrm{in}}/\epsilon_{\mathrm{out}}$, α=0.571412, and *A* is the electrostatic size of the molecule, which is essentially the overall size of the structure that can be computed analytically. We employ the most widely used functional form $f_{ij}^{\mathrm{GB}}={\left[r_{ij}^{2}+R_{i}R_{j}\exp(-r_{ij}^{2}/4R_{i}R_{j})\right]}^{\frac{1}{2}}$, where $r_{ij}$ is the distance between atomic charges $q_{i}$ and $q_{j}$, and $R_{i}$, $R_{j}$ are the so-called *effective Born radii* of atoms *i* and *j*, which represent each atom’s degree of burial within the solute. The effective Born radii, *R*, are calculated by the “$R^{6}$” equation [60,61]

(7) $$\begin{array}{c} R_{i}^{-3}=\left(-\frac{1}{4\pi }\oint_{\partial V}\frac{r-r_{i}}{|r-r_{i}{|}^{6}}\cdot dS\right), \end{array}$$

where ∂V indicates the chosen representation of the dielectric boundary of the molecule, dS is the infinitesimal surface element vector, $r_{i}$ is the position of atom *i*, and r represents the position of the infinitesimal surface element. Uniform offset to the inverse effective radii is set to the default (optimal) value of 0.028 Å${}^{-1}$ [62]. The screening effect of monovalent salt is introduced into Equation (6) as is standard for the GB model [53]; in our MM/GBSA calculations the salt concentration was set to 0.1 M.

#### 2.3.2. Non-polar Component

A common method to estimate the non-polar contribution to the solvation free energy in Equation (3) is to assume that it is proportional to the solvent-accessible surface area (SASA) of the molecule

(8) $$\begin{array}{c} G_{nonpol}=\gamma *SASA. \end{array}$$

While there are more accurate methods to estimate the non-polar [63] contribution, here we use the simple Equation (8) for the sake of simplicity and consistency with [28]. For consistency with the same work, here we use γ=0.0072 kcal/mol/A${}^{2}$. Atomic radii that form SASA not only play an important role in the non-polar component, but also enter the polar component through the dielectric boundary. Therefore, the right choice of atomic radii is crucial to the accuracy of binding free energy estimation [64,65]. Two sets of atomic radii are used here: global optimal radii for $\Delta G_{bind}$ calculations (OPT1) [43,44] and Bondi [66], see Table 1. The van der Waals radii determined by Bondi from molecular crystals and noble gas crystals are commonly known as “all-purpose” sets of intrinsic atomic radii in a wide range of molecular modeling applications [67] including continuum solvent calculations [68]. OPT1 radii were specifically optimized to best reproduce the explicit solvent results, particularly in the implicit solvent-based binding MM/GBSA estimates. Carbon (C), hydrogen (H), oxygen (O), nitrogen (N), and sulfur (S) are the main atomic types in this study. The water probe radius is fixed to 1.4 Å. Note that OPT1 radii have been optimized only on C, H, O, and N. The remaining atomic radii are identical to Bondi radii.

### 2.4. Gas-Phase Energy

Gas-phase energy of the solute, $\Delta E_{MM}$, is the summation of internal energies, electrostatic energies, and van der Waals energies. In all of the MM/GBSA calculations reported here, $\Delta E_{MM}$ is calculated using the ff99 AMBER force field. The choice of this old force field is deliberate, and was initially motivated to ensure maximum consistency with [8], which provides a very detailed analysis of MM/GBSA performance on Ras–Raf. Good agreement with experiment motivated us to use the same ff99 force field for all the subsequent MM/GBSA calculations reported here. All of the enthalpy calculations in this study are averages over 500 snapshots extracted from the main MD trajectory.

### 2.5. Configurational Entropy

NMA is selected for entropy calculations according to its promising convergence compared to other methods, such as quasi-harmonic analysis [28]. The main drawback of this method is the computational cost, which becomes intractable for large systems, e.g., systems with more than 8000 atoms in MM/GBSA (Perl version) of AMBER18 are not supported for NMA. To tackle this problem, one standard approach is to truncate the complex so that the binding interface is preserved in its original shape [40]. In this study, a novel truncation algorithm is proposed and tested on the Ras–Raf complex. Encouraged by the accurate results, the SARS-CoV-2 S RBD and ACE2 complex is truncated based on a similar algorithm for NMA feasible calculations. An offset of 1.92 kcal/mol has been subtracted from the −TΔS component of GBNSR6 calculations to address the concentration- dependency of the translational entropy at 1 M, see [28] for details. NMA entropy calculations are done over 150 snapshots extracted from the main MD trajectory unless stated otherwise.

### 2.6. Structure Preparation

#### 2.6.1. Ras–Raf Complex

This well-studied complex was selected as the reference for testing the parameters of the MM/GBSA model and the proposed truncation algorithm. We used tleap module in AMBER18 to set up the input coordinate and topology files. The structure was solvated in a box of TIP3P [69] water model (10 Å buffer). This choice of old water model and ff99 AMBER force field was intended to ensure full consistency with [8]. The GTP molecule and the magnesium ion (Mg${}^{2+}$) were eliminated for the sake of simplification. No counterions were added to the system.

#### 2.6.2. SARS-CoV-2 S RBD and ACE2 Complex

H++ server [70] was employed to protonate the complex (PDB ID: 6M0J) at pH = 7.5. The server automatically generates the solvated structure in a box of OPC [71] explicit water model (10 Å buffer), with the AMBER ff14SB force field. This full structure is used only for enthalpy calculations that are compared with those of the truncated complex structure for justifying the truncation approach, see below.

### 2.7. MD Simulation

All of the MM/GBSA estimates are based on snapshots extracted from MD trajectories, generated as described below. The solvated complexes were first energy-minimized (max. minimization cycle of 1000), followed by 50 ps of heating (from 1 K to 300 K) at constant volume, followed by 50 ps of density equilibration at 300 K at constant 1 bar pressure, followed by another 2 ns of constant (1 bar) pressure equilibration at 300 K. In these stages, atomic coordinates were restrained to their initial positions with 2 kcal/mol/A${}^{2}$. All simulations, including the production runs described below, were executed with the GPU-enabled pmemd.cuda MD engine in AMBER18, using Langevin dynamics with a collision frequency of 2 ps${}^{-1}$ and an integration time step of 2 fs, while the bonds involving hydrogen atoms were constrained by the SHAKE algorithm. Electrostatic interactions were approximated via the Particle Mesh Ewald (PME) method, with a non-bond cutoff set to 9 Å. Coordinates were recorded every 10 ps. A production of 10 ns was performed using the Ras–Raf structure prepared with the protocol described in Section 2.6. A production of 50 ns was carried out using the SARS-CoV-2 S RBD and ACE2 complex structure described in Section 2.6.

### 2.8. Proposed Truncation Algorithm

As an improvement to the standard truncation methods [40], the following algorithm is suggested for addressing the computational inefficiency of entropy calculation via NMA: first, the binding interface of the given complex is identified via visualization of the structure and locating receptor and ligand residues that are around 8 Å from another. Residues that are out of this range are candidates to be eliminated from the ligand terminal by trimming its amino acid sequence. Depending on the size of the structure, it might be necessary to eliminate residues from the protein terminal to enable NMA calculations. In this algorithm, the final truncated structure will be one connected component ,which is easier to interpret compared to the outcome of standard truncation methods [40]. Specifically, this new characteristic reinforces control over the motion of residues in the binding interface during the MD simulation. Based on the protocol used for the full structure, MD simulation of the truncated structure is carried out. A weak restraint of 0.01 kcal/mol/A${}^{2}$ is applied to the atoms of the truncated complex relative to the X-ray positions during the production, preventing the truncated complex from falling apart.

## 3. Results and Discussion

### 3.1. Efficient Calculation of Entropy: Continuous Truncation of Protein Structures

#### 3.1.1. NMA on the Truncated Ras–Raf Complex

**Figure 5:**
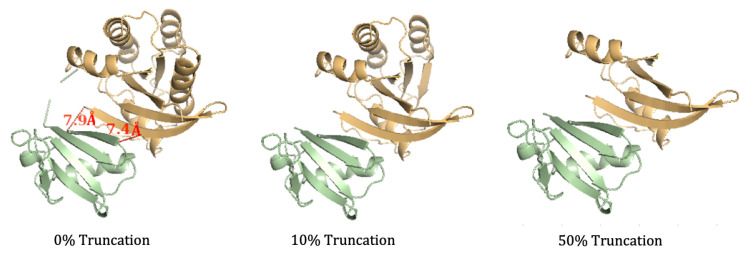
Truncated structures of the Ras–Raf complex. The protein, Ras, is shown in orange and the ligand, Raf, is shown in green. The untruncated complex (full structure) is on the left, followed by the 10% truncated structure (17 residues of Ras eliminated) in the middle, and the 50% truncated structure (83 residues of Ras eliminated) on the right. The image on the left shows two pairs of residues in the binding interface that are less than 8 Å apart.

To test the accuracy of the proposed truncation algorithm, NMA entropy was calculated on three truncated structures of the Ras–Raf, as follows: the binding interface of the complex was found through visualization, and the residues of the Ras protein within 8 Å of the Ras ligand were considered necessary for calculation, as removal within this distance would minimize absolute entropy changes [40]. In these modifications, only the protein structure was modified as it was not necessary to modify the ligand to decrease the duration of the NMA calculation. The binding interface of the complex was found to be residues 36–41 Ras, and this region remained conserved in all truncated simulations. Three simulations were run: 0% truncation (the full structure of the complex), 10 % truncation, and 50% truncation. In each of the scenarios, residues were removed one-by-one from the PDB files to create continuous sequences with the appropriate truncation. For the 0% truncation MD simulation, no residues were removed. For the 10% truncation, the last 17 residues of Ras were removed (150–166), and for the 50% truncation, the last 83 of the residues (84–166) in Ras were removed. The truncated structures are shown in Figure 5. The production file for the MD simulation was modified to account for the restraints necessary for the truncation. Only two snapshots were taken from the NMA (at the beginning and the end) to retrieve the entropy values. After adjusting for concentration dependence, each of the three simulations gave a −TΔS value of 37.26 ± 3.21 kcal/mol. These results indicate that so long as the binding interface remains conserved and the structures are continuous, truncating protein structures do not impact the validity of NMA calculations.

#### 3.1.2. NMA on the truncated SARS-CoV-2 S RBD and ACE2 Complex

**Figure 6:**
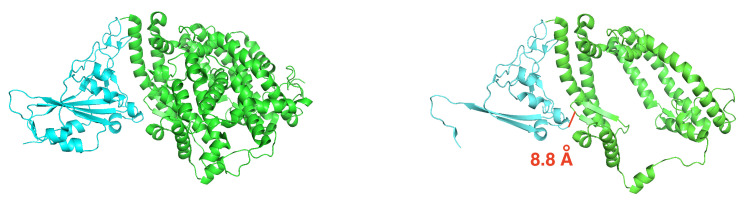
Truncation of SARS-CoV-2 S RBD used in the entropy estimate. The spike protein is in cyan, and the ACE2 receptor is in green. Left: original complex. Right: truncated complex. A pair of atoms on the binding interface that are 8.8 Å apart is shown in a solid red segment to illustrate the length scale.

To execute NMA entropy calculations, the original structure of SARS-CoV-2 S RBD bound with ACE2 (PDB ID: 6M0J) was truncated from 12,515 atoms (791 residues) to 7286 atoms (463 residues) by removing residues, one by one, starting from the N-terminus of the spike protein, and the C-terminus of the ACE2 protein. The goal was to have fewer than 8000 atoms remaining while preserving sequence continuity of the resulting structure to facilitate the set-up of MD simulations. Figure 6 shows this truncation. The remaining atoms are still within 8 Å from the binding interface. The same protocol used for the full structure was employed for parameterization and solvation.

**Figure 7:**
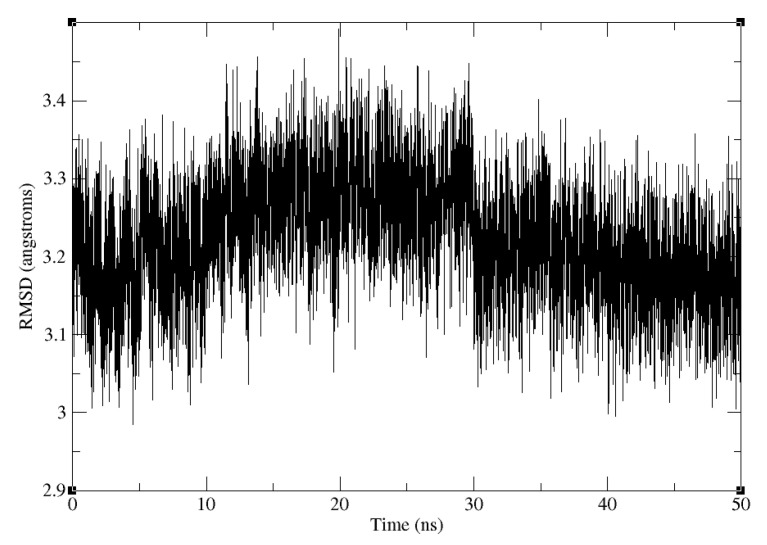
Backbone RMSD of the truncated SARS-CoV-2 S RBD and ACE2 complex relative to the truncated part of the experimental crystal structure of the full complex, along the 50 ns production trajectory.

The RMSD of the truncated SARS-CoV-2 S RBD and ACE2 backbone compared to the crystal structure of the full complex is shown in Figure 7. The trajectory is stable after 50 ns of production, with the RMSD from the X-ray reference of around 3.15 Å(the RMSD convergence was checked by running the next 50 ns of the MD simulation). The weak restraint on the truncated structure diminishes the discrepancy between the force field and water model in the structure used for MD simulation (OPC, ff14SB) and the one for $\Delta G_{bind}$ calculations (TIP3P, ff99).

### 3.2. Efficient Calculation of Entropy: Selection of a Few Snapshots

**Figure 8:**
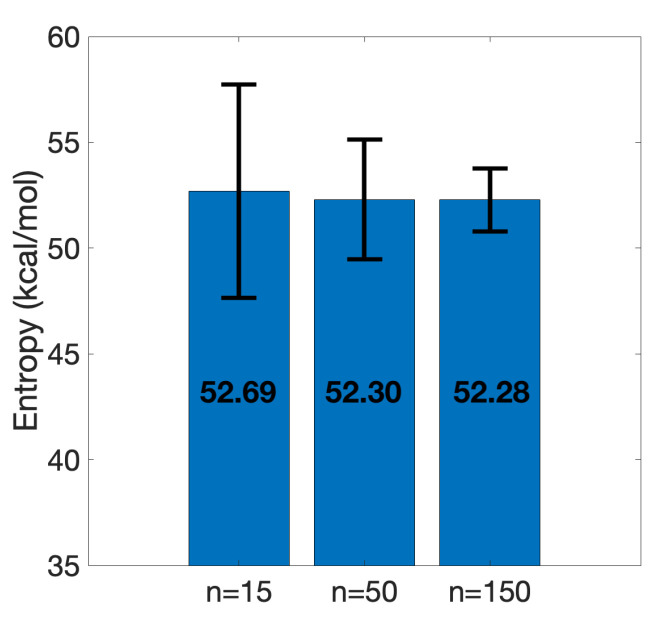
Entropy convergence of the truncated SARS-CoV-2 S RBD and ACE2 complex. Means and standard error of the means are shown. Increasing the number of equidistant sample points from 15 to 150 shows the stability of the entropy around 52 kcal/mol.

Entropy calculation is one of the most challenging and time-consuming parts of $\Delta G_{bind}$ estimation. Even after truncation, it took one day using 10 threads in parallel to calculate −TΔS on 150 snapshots on a server with the following specifications: Intel (R) Xeon (R) CPU 2.60 GHz and 32 GB RAM. In order to maintain a consistent protocol, 150 snapshots were selected for −TΔS calculations. We also conducted an investigation to examine whether a fewer number of snapshots would be sufficient for accurate calculation of entropy. Two subsets, one of 15 equidistant snapshots and the other of 50 equidistant snapshots, were collected from the set of 150 snapshots. According to Figure 8 it is observed that entropies calculated on 15 and 50 sample snapshots lead to a similar −TΔS calculated on the whole set. Apparently, the standard error of the mean decreases as the sample size increases; however, this does not affect the stability of the mean around 52 kcal/mol. Given entropy calculation as the bottleneck of more accurate $\Delta G_{bind}$ estimations, this observation suggests that with a relatively small set of snapshots, it is still possible to accurately compute −TΔS.

### 3.3. MM/GBSA on Ras–Raf

**Figure 9:**
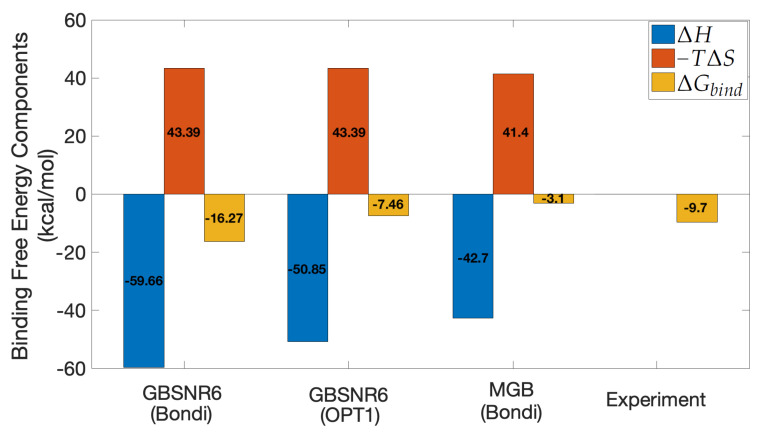
MM/GBSA results for Ras-Raf. The experimental value is from isothermal titration calorimetry [72]. An offset of 1.79 kcal/mol has been subtracted from the ΔH component of MGB-based estimate [28] for consistency with the author’s recommendation.

GBNSR6—the most accurate flavor of GB in terms of calculating polar binding free energy [56]—was selected as the implicit solvent model in MM/GBSA. In this model, the choice of atomic radii plays an important role, reflected through the dielectric boundary in $\Delta G_{pol}$ and SASA in $\Delta G_{nonpol}$. Two sets of atomic radii are considered here: the standard Bondi radii and the recently optimized radii set called OPT1. According to Figure 9, it is observed that $\Delta G_{bind}$ calculated by GBNSR6 coupled with OPT1 radii underestimates the binding affinity, whereas GBNSR6 coupled with Bondi radii overestimates it. One rationale for this observation would be that Bondi and OPT1 have very different physical foundations behind them (geometry for the former and global optimization of the electrostatics for the latter), so the resulting errors are not as strongly correlated as for radii derived on the same principle. Both of these results have better agreement with the experiment [72] compared to the reference MGB model in [28]. It is noticed that the consensus estimate $\Delta G_{bind}=\left(G_{bind(Bondi)}+G_{bind(OPT1)}\right)/2\approx -11.87$ kcal/mol is only 2 kcal/mol off the experimental reference. Encouraged by this agreement with experiment, we used GBNSR6 with both Bondi and OPT1 radii to produce a consensus estimate of $\Delta G_{bind}$ for the SARS-CoV-2 S RBD and ACE2 complex.

### 3.4. MM/GBSA on SARS-CoV-2 S RBD and ACE2

The result of calculating $\Delta G_{bind}$ on the truncated structure (explained in Section 3.1) and the full structure (explained in Section 2.6) are shown in Table 2. The entropy term of the both structures is the same, as expected from the truncation process explained in Section 3.1. The enthalpy term, however, is calculated on the truncated and full structures separately. The final estimates are compared to another study [73], in which $\Delta G_{bind}$ of a structure similar to the SARS-CoV-2 S RBD and ACE2 complex has been determined experimentally. Similar to Figure 9, it is observed that the two radii sets provide a feasible range in which the experimental value lays. More specifically, $\Delta G_{bind}$ calculated with Bondi radii provides a lower bound and $\Delta G_{bind}$ calculated by OPT1 radii provides an upper bound on the binding affinity of the structure. Incidentally, the consensus estimate for the truncated and full complexes are $\Delta G_{bind}=-9.4\pm 1.5$ kcal/mol and $\Delta G_{bind}=-2.92\pm 1.5$ kcal/mol, respectively. The former is in near quantitative agreement with the experiment, suggesting that working with the truncated structure not only accelerates the computations, but also leads to a more accurate estimation of absolute binding free energy. The consensus estimate of the full structure, however, is quite off the experimental reference. In particular, $\Delta G_{bind}$ calculated with the OPT1 atomic radii is extremely underestimated which may necessitate further investigations in radii optimization. This observation could also be due to the exact replication of entropy term for the truncated and full structures. A more accurate alternative could be finding a constant factor (or a function) that adjusts the entropy term adapted from the truncated structure to the original structure.

## 4. Conclusions

In this study, an effective MM/GBSA protocol is introduced for the absolute binding free energy calculation of SARS-CoV-2 S RBD and ACE2 complex. This protocol is designed based on the Ras–Raf complex, which has a similar energy profile and has been widely studied in the literature. We evaluated the performance of a relatively new GB model and the newly introduced intrinsic atomic radii, called OPT1, in binding free energy calculation of Ras–Raf. A common set of atomic radii (Bondi) was also tested. It was observed that the two radii sets provide a reasonable range for $\Delta G_{bind}$, which contains the experimental value. More specifically, $\Delta G_{bind}$ calculated with Bondi radii is overestimated (lower bound), whereas $\Delta G_{bind}$ calculated by OPT1 radii is underestimated (upper bound). The consensus estimate of the two $\Delta G_{bind}$s (i.e., the midpoint of the range) was in quantitative agreement with the reference experiment. Encouraged by the better agreement with experiment for Ras–Raf compared to a previous work, we applied the same approach to estimate $\Delta G_{bind}$ of the SARS-CoV-2 S RBD and ACE2 complex. As a bottleneck of our simulation, entropy calculation was studied exclusively. We modified the standard truncation approach for dealing with large structures so that the final truncated structure becomes one connected component which is biologically interpretable. In order to confirm that working with the truncated structures does not affect the accuracy of final $\Delta G_{bind}$, the enthaply component was calculated for both full and truncated structures of SARS-CoV-2. Similar to the Ras–Raf case study, it was observed that the $\Delta G_{bind}$ calculated by the two sets of radii provide a feasible range in which the consensus estimate demonstrates quantitative agreement with the experiment for the truncated structure. The high binding affinity of SARS-CoV-2/ACE2, which has been experimentally measured and computationally validated, may be associated with the great severity of the virus. The proposed MM/GBSA protocol is recommended for future analysis of *relative* binding free energies in the SARS-CoV-2 S RBD and ACE2 system, including the effects of mutations, relative contributions from various residues to $\Delta G_{bind}$ and congeneric series of ligands.

## Figures and Tables

**Table 1:** Two sets of atomic radii in Å used in this study.

	$\rho_{C}$	$\rho_{H}$	$\rho_{N}$	$\rho_{O}$	$\rho_{S}$	$\rho_{F}$	$\rho_{\mathrm{Cl}}$	$\rho_{I}$
Bondi	1.70	1.20	1.55	1.52	1.80	1.47	1.75	1.98
OPT1	1.40	1.55	2.35	1.28	1.80	1.47	1.75	1.98

**Table 2:** MM/GBSA results on the truncated SARS-CoV-2 S RBD and ACE2 complex. Means and the standard errors of the mean are listed. All the components are in kcal/mol. Experimental value is derived from a fit to surface plasmon resonance sensogram [73].

	Truncated Structure		Full Structure		Exp.
	**Bondi**	**OPT1**	**Bondi**	**OPT1**	
$\Delta E_{MM}$	−453.76 ± 0.87	−453.76 ± 0.87	−614.29 ± 0.91	−614.29 ± 0.91	
$\Delta G_{nonpol}$	−14.71 ± 0.02	−16.35 ± 0.02	−14.32 ± 0.01	−16.42 ± 0.02	
$\Delta G_{pol}$	401.55 ± 0.81	413.72 ± 0.87	566.49 ± 0.84	582.43 ± 0.87	
ΔH	−66.93 ± 0.29	−56.39 ± 0.37	−62.12 ± 0.31	−48.28 ± 0.44	
−TΔS	52.28 ± 1.49	52.28 ± 1.49	52.28 ± 1.49	52.28 ± 1.49	
$\Delta G_{bind}$	−14.65 ± 1.52	−4.11 ± 1.54	−9.84 ± 1.52	4 ± 1.55	−10.6

## Data Availability

Truncated structures and MD inputs have been available since 18 September 2020, on https://github.com/NeginForouzesh/sars-cov-2-spike-and-human-ace-2. MD trajectories can be found on the COVID-19 Molecular Structure and Therapeutics Hub via https://covid.molssi.org/simulations/.
